# A Splice Variant of Bardet-Biedl Syndrome 5 (BBS5) Protein that Is Selectively Expressed in Retina

**DOI:** 10.1371/journal.pone.0148773

**Published:** 2016-02-11

**Authors:** Susan N. Bolch, Donald R. Dugger, Timothy Chong, J. Hugh McDowell, W. Clay Smith

**Affiliations:** Department of Ophthalmology, University of Florida, Gainesville, Florida, United States of America; National Eye Institute, UNITED STATES

## Abstract

**Purpose:**

Bardet-Biedl syndrome is a complex ciliopathy that usually manifests with some form of retinal degeneration, amongst other ciliary-related deficiencies. One of the genetic causes of this syndrome results from a defect in Bardet-Biedl Syndrome 5 (BBS5) protein. BBS5 is one component of the BBSome, a complex of proteins that regulates the protein composition in cilia. In this study, we identify a smaller molecular mass form of BBS5 as a variant formed by alternative splicing and show that expression of this splice variant is restricted to the retina.

**Methods:**

Reverse transcription PCR from RNA was used to isolate and identify potential alternative transcripts of Bbs5. A peptide unique to the C-terminus of the BBS5 splice variant was synthesized and used to prepare antibodies that selectively recognized the BBS5 splice variant. These antibodies were used on immunoblots of tissue extracts to determine the extent of expression of the alternative transcript and on tissue slices to determine the localization of expressed protein. Pull-down of fluorescently labeled arrestin1 by immunoprecipitation of the BBS5 splice variant was performed to assess functional interaction between the two proteins.

**Results:**

PCR from mouse retinal cDNA using Bbs5-specific primers amplified a unique cDNA that was shown to be a splice variant of BBS5 resulting from the use of cryptic splicing sites in Intron 7. The resulting transcript codes for a truncated form of the BBS5 protein with a unique 24 amino acid C-terminus, and predicted 26.5 kD molecular mass. PCR screening of RNA isolated from various ciliated tissues and immunoblots of protein extracts from these same tissues showed that this splice variant was expressed in retina, but not brain, heart, kidney, or testes. Quantitative PCR showed that the splice variant transcript is 8.9-fold (+/- 1.1-fold) less abundant than the full-length transcript. In the retina, the splice variant of BBS5 appears to be most abundant in the connecting cilium of photoreceptors, where BBS5 is also localized. Like BBS5, the binding of BBS5L to arrestin1 can be modulated by phosphorylation through protein kinase C.

**Conclusions:**

In this study we have identified a novel splice variant of BBS5 that appears to be expressed only in the retina. The BBS5 splice variant is expressed at approximately 10% of full-length BBS5 level. No unique functional or localization properties could be identified for the splice variant compared to BBS5.

## Introduction

In cells with a sensory cilium, the cilium functions as a probe for the cell’s environment, sensing external physiological, chemical, and physical cues, and then transducing this information internally to the cell for the appropriate response [1]. The importance of cilia is reflected in the large array of diseases that are a consequence of ciliary defects, such as retinal degeneration, deafness, anosmia, obesity, and mental retardation [2,3].

The outer segment of photoreceptors is an extreme example of a highly modified sensory cilium adapted for transducing light into a change in membrane potential. Consistent with other non-motile sensory cilia, the outer segment cilium originates from a basal body from which extend nine doublets of microtubules that extend through the transition zone, often referred to as the “connecting cilium” [4]. In contrast to other cilia, however, the ciliary membrane in photoreceptors is highly developed, forming a series of stacked lamellae (in cones) or stacked discs (in rods) that contain a high concentration of visual pigment molecules for capturing photons.

The development and maintenance of this highly specialized structure is dependent upon a carefully regulated process which allows entry of elements that belong in the outer segment while at the same time excludes elements that do not belong in the outer segment. One of the elements that is involved in this regulatory process is the BBSome, a complex of seven proteins that is important in regulating the protein composition in all cilia, including photoreceptor outer segments [5–8]. Not surprisingly, defects in the BBSome elements often result in ciliary deficits which are manifested as the ciliopathy known as Bardet-Biedl Syndrome [9,10].

In photoreceptors, the BBSome currently has two known roles. First, the BBSome appears to function through interaction with Rab8 as a key regulator in vesicle trafficking from the Golgi to the base of the cilium [7,8,11]. The second role for the BBSome appears to be as an adaptor molecule for cargo transport along the cilia via the intraflagellar transport pathway based on conservation of function with other ciliary systems [12–15]. In photoreceptors, defects in BBSome components lead to disrupted outer segment development and opsin mislocalization, resulting in defects in photoreceptor functionality and degeneration [16–18].

In addition to these functions, it appears that some elements of the BBSome may have additional roles. For example, BBS5 was recently shown to localize along the axoneme of the photoreceptor where it regulates binding of arrestin1 in a light-dependent manner [19]. In this study, we extend an observation we made as part of our studies of BBS5 in which we noted an apparently smaller BBS5-like protein based on immunoreactivity. This study identifies the smaller BBS5 protein as a splice variant of BBS5 and provides initial characterization of this novel protein.

## Materials and Methods

### Animal Welfare

All animal work was conducted according to the Association for Research in Vision and Ophthalmology guidelines for the use of animals in ophthalmic and visual research under the approval of the University of Florida’s Institutional Animal Use and Care Committee.

### Human Tissue

Human neural retina used in this study was obtained from the Lions Eye Institute for Transplant and Research, Inc. The Lions Eye Institute obtains and maintains research consent records for all donated tissue.

### Retinal Extracts

Retinas from cow (*Bos taurus*), mouse (*Mus musculus* C57BL/6J), rabbit (*Oryctolagus cuniculus*), frog (*Xenopus laevis*), human (*Homo sapiens*), and pig (*Sus scrofa*) were homogenized in 50 mM HEPES (pH 7.4) with 200 mM NaCl, 1 mM EGTA, 1 mM MgCl_2_, 10% glycerol, and 0.05% NP-40, incubated on ice for 60 min, and then centrifuged (30 min, 30,000 x g) to obtain a cleared supernatant. Extracts from mouse brain, heart, kidney, and testis were prepared using the same methodology. The protein concentration of each extract was determined (MicroBCA Assay, Pierce) and 10 μg of extract from each species loaded on 12% sodium dodecyl sulfate polyacrylamide gels for electrophoresis under denaturing conditions. Separated proteins were transferred to polyvinylidene fluoride membrane (Immobilon-P, GE Healthcare Life Sciences). Immunoblots were probed with anti-BBS5 #7–15 monoclonal antibody (described in [19]) and detected using chemiluminescence (Western Breeze, Life Technologies). Replicate blots were probed with anti-β-tubulin monoclonal antibody [20] as a quality control.

### cDNA Synthesis

Polyadenylated RNA (polyA^+^) was isolated from retinal or other mouse organ homogenates in guanidine thiocyanate (QuickPrep Micro mRNA Purification, GE Healthcare Life Sciences) and reverse transcribed after priming with oligo(dT) (FirstStrand cDNA Synthesis, GE Healthcare Life Sciences). In some cases, the 5’ end of the Bbs5 RNA was selectively extended and a 5’ anchor priming site generated (5’ RACE System, Life Technologies). In these instances, primers against exons 1, 5, and 9 were used to prime cDNA synthesis. Polymerase chain reaction (PCR) was performed on this cDNA with Taq polymerase (GoTaq, Promega) and primer pairs directed against all possible combinations of the represented exons (Table 1); cDNA was amplified with primers directed against α-tubulin for quality control. Reactions were separated by horizontal agarose electrophoresis through 1.2% agarose. For sequencing, amplified DNA was excised for extraction (Gel Extraction, Qiagen), ligated into pCR2.1 (Life Technologies), and colonies selected for dideoxy chain termination sequencing. For identification of potential additional transcripts, primers were designed that either flanked the predicted deleted exon, or were part of an included intron in the alternative transcript (Table 2).

**Table 1:** Oligonucleotide primers used for cDNA synthesis and PCR.

Primer	Sequence	Location on *Bbs5* gene	Direction
5’R	GGCCACGCGTCGACTAGCACGGGIGGGIIGGGIIG	5’ RACE primer	sense
A1	GAGTCTACCTCTGTCTCCATTGTTTCC	Exon 1	anti
A2	GCTGCTTGTTCTGAATGACTGCACTTCTC	Exon 5	anti
A3	GCCTCGAGCCTCAGCTGCTTGTTCTGAATG	Exon 6	anti
A4	GCCTCGAGGCGCCAAACCAAATTTTGAATCTC	Exon 7	anti
A5	GCCTCGAGGGCTGTGGCTTTTCTTCCATTTC	Exon 9	anti
S1	GCCTCGAGGATCAATGGCGTATGGAATCTGTCC	Exon 6	sense
S2	GTGGCATGCCAATATGAATGACAG	Exon 7	sense
S3	GCCTCGAGCTGTGGAAAAACTACAGGAATCAG	Exon 8	sense
S4	GCTTTTGTGGCGTATTTTGCTGATGGG	Exon 9	sense
S5	GCTTTTGTGGCGTATTTTGCTGATGGG	Exon 10	sense
qPCR1	GCGAATTCTCAACAGGGTTTCTGACTTTCACAC	Exon 7a	anti
qPCR2	CTGGCAGAATAGACTTTGTGAAGTGAG	Exon 9	anti
dT	AACTGGAAGAATTCGCGGCCGCAGGAATTTTTTTTTTTTTTTTTT	poly(A) tail	anti
α-tub1	GTACGCGTCCATGCGTGAGTGCATCTCCATCCAC	α-tubulin 5’	sense
α-tub2	GCACGCGTTTAGTACTCCTCTCCTTCTTCCTC	α-tubulin 3’	anti

**Table 2:** Alternative transcripts for murine Bbs5 predicted in the Ensembl and MGI databases, and the oligonucleotide primers designed to identify these potential transcripts.

Transcript (database)	Variation	Primer Pair	Predicted PCR product (normal; alternative transcript)
Bbs5-002 (Ensembl)	Exon 5 deleted	ATGTCTGTGCTGGACGTGTTGTGG CCTCAGCTGCTTGTTCTGAATG	485 bp; 357 bp
Bbs5-003 (Ensembl)	Exon 8 deleted	GTGGCATGCCAATATGAATGACAG CTGGCAGAATAGACTTTGTGAAGTGAG	216 bp; 152 bp
Bbs5-004 (Ensembl)	Retained Intron 2	ATGTCTGTGCTGGACGTGTTGTGG CCACTGAACCATCTCACCAGCCC	--; 240 bp
Bbs5-005 (Ensembl)	Retained Intron 4	ATGTCTGTGCTGGACGTGTTGTGG GTTTCAGAGTTAAAGAGCAAGTTCCC	--; 350 bp
Bbs5-006 (Ensembl)	Retained Intron 3	ATGTCTGTGCTGGACGTGTTGTGG CTCTACCATCCAACTTTCCTG	--; 280 bp
Bbs5-007 (Ensembl)	Retained Intron 5	GGATTTTAACACAGCCTAACAAACCAC CCTCAGCTGCTTGTTCTGAATG	--; 135 bp
Bbs5-008 (Ensembl)	Exon 9 deleted	GATCAGAGATTCAAAATTTGGTTTGGCGC CCCAGTTCTTCTGAAAATACAGGTTC	320 bp; 190 bp
Bbs5-X1 (MGI)	Retained Intron 7	GTGGCATGCCAATATGAATGACAG GCCTTGACTCCTCCACAGTCCACAGG	--; 206 bp
Bbs5-X2 (MGI) (= Bbs5L)	Retained Intron 7	GTGGCATGCCAATATGAATGACAG CTGGCAGAATAGACTTTGTGAAGTGAG	216 bp; 379 bp

-- indicates no product expected for normal transcript

### Quantitative PCR

cDNA prepared from poly(A)^+^ RNA isolated from whole mouse retina and primed with oligo(dT) was used for quantitative real-time PCR. Real-time PCR was performed on an iCycler (Bio-Rad) using iQTM Syber Green Supermix (Bio-Rad), with threshold cycle number (Ct) determined by the cycler software and optimal primer concentrations determined separately for each primer pair. To test primer efficiencies, one-step reverse transcriptase PCR was run with each target primer. Primers S2 and qPCR1 (Table 1) were used to selectively amplify the splice variant cDNA, and primers S2 and qPCR2 (Table 1) were used to amplify Bbs5. Three dilutions of each of three separate template retinal cDNA were prepared for each primer pair. Relative amounts of splice variant product was calculated in comparison to the Bbs5 product by the 2^-ΔΔCt^ method using β-actin as the reference control [21] and statistically compared using Student’s t-test. Samples were tested for purity of the amplified product by electrophoreses through 1.2% agarose gel.

### Protein Expression and Purification

cDNA for Bbs5 and the splice variant of Bbs5 (Bbs5L) were amplified from murine retinal cDNA with primers that incorporated *Eco*RI sites flanking the open-reading frames. These cDNA’s were cloned into pET-28a at the *Eco*RI site, and transformed into *Escherichia coli* strain BL21(DE3). Expression was induced with 30 μM IPTG overnight at 37°C, bacteria harvested, and lysed in a French press at 20,000 psi in 50 mM sodium phosphate buffer with 300 mM sodium chloride and 10 mM imidazole (pH 7.0). Inclusion bodies in the pellet were solubilized in the same phosphate buffer with 6 M guanidine hydrochloride overnight with constant rotation (4°C). A cleared supernatant was prepared (30,000 x g, 30 min) and purified over nickel agarose (His GraviTrap; GE Healthcare Life Sciences). Protein was eluted with a gradient of 10–500 mM imidazole in phosphate buffer with guanidine HCl, and dialyzed against phosphate-buffered saline with multiple changes to remove the guanidine HCl.

For experiments in which the recombinant BBS5 protein was used to pre-absorb the anti-BBS5 antibody, 10 mg of BBS5-containing inclusion bodies were resuspended with the monoclonal anti-BBS5 #7–15 antibody [19], incubated at room temperature for 60 min, and then centrifuged (16,000 x g) for 10 min to obtain a cleared supernatant that was used for immunoprobing.

### Arrestin1 binding assay

To test binding of arrestin1 to BBS5L and BBS5 we prepared Bbs5L and Bbs5 in fusion with glutathione-S-transferase (GST). cDNAs for His-tagged BBS5L and BBS5 were cloned into pGEX-4T-1 at the *Eco*RI site, generating a fusion protein with the C-terminus of GST. Proteins were expressed in NEB 5-alpha *E*. *coli* (New England Biolabs) in Luria-Bertani broth containing 30 μM IPTG, with protein purification as described above. Following purification, the BBS5L-GST and BBS5-GST proteins were refolded and an aliquot of each phosphorylated by protein kinase C (PKC) as previously described [19]. To verify that BBS5L and BBS5 were phosphorylated, samples of the proteins treated with PKC were immunoblotted and probed with anti-phosphoserine antibody (Life Technologies). Purification of the sample was estimated at >95% by SDS-PAGE by staining electrophoresed samples with Coomassie blue. Protein concentration was determined spectrophotometrically. Fluorescently labeled arrestin1 was prepared using I16C-substituted bovine arrestin1 to generate a reactive sulfhydryl group that was labeled with AlexaFluor-546 [22]. GST used as a control for non-specific binding was expressed using the pGEX-4T-1 vector and purified over a GST GraviTrap column (GE Healthcare Life Sciences).

For the binding assay, 6.6 μM I16C-Alexa546-ARR1 was mixed with 0.1 μM BBS5L-GST, BBS5-GST, or GST and allowed to incubate overnight at 4°C. The mixture was then immunoprecipitated with anti-GST antibody (Rockland Inc) conjugated to magnetic beads (DynaBeads, Life Technologies) and fluorescence of the captured arrestin1 measured as previously described [19]. Samples were prepared in duplicate and the assay repeated three separate times. Samples were statistically compared using Student’s t-test.

### Immunohistochemistry

To generate antibodies specific for BBS5L, a 24 amino acid peptide corresponding to the unique C-terminus of BBS5L was synthesized and conjugated to bovine serum albumin (EPRRTLAPHKWKIVSLLCESQKPC-BSA; Genscript). New Zealand White rabbits were immunized with 50 μg/mL peptide in phosphate-buffered saline with Sigma Adjuvant System oil (Sigma-Aldrich), boosted at two week intervals, and serum harvested at 12 weeks. Immunoglobulins were isolated from the serum by affinity purification (Protein A/G column; Thermo Scientific). Specificity of the anti-serum for BBS5L was tested against heterologously expressed BBS5L compared to BBS5 as described in the results. In some cases, BBS5L anti-serum was conjugated directly to AlexaFluor-647 for direct detection. In this case, BBS5L anti-serum was pre-labeled with AlexaFluor-647 succinimidyl ester (AlexaFluor-647 Protein Labeling kit, Life Technologies), and purified over a size-exclusion column to remove unreacted label.

For tissue staining, whole mouse eyes (C57BL/6J) were cryofixed in liquid nitrogen-chilled isopentane without chemical fixation [23], embedded in optimal cutting tissue compound (Tissue-Tek, VWR Scientific), and cryosections cut at 12 μm thickness. Sections were blocked with 2% ultra-low immunoglobulin fetal bovine serum (Life Technologies) with 0.1% Triton X-100. Slides were then serially stained with anti-BBS5 #7–15 monoclonal antibody [19] and anti-retinitis pigmentosa 1 (RP1) antibody (a kind gift from Eric Pierce, [24]) overnight (room temperature), followed by anti-mouse secondary antibody conjugated to AlexaFluor-488 and anti-chicken secondary antibody conjugated to Texas Red with 10 μg/mL 4',6-diamidino-2-phenylindole (2 h, room temperature, Life Technologies), and finally with anti-BBS5L-Alexa 647 conjugate (2 h, room temperature). At each step, slides were extensively washed with 0.1% Triton X-100 in phosphate-buffered saline. Sections were imaged with an UltraVIEW VoX, 3D spinning disk laser confocal microscope, equipped with a Yokogawa CSU-X1 spinning disk scanner (PerkinElmer), and images processed with Volocity 3D Imaging software (PerkinElmer). To demonstrate specificity of the anti-sera, sections were also probed with anti-sera that were preabsorbed to recombinantly expressed BBS5 protein (for BBS5 #7–15 monoclonal) or against recombinant BBS5L protein (for anti-BBS5L-Alexa 647 conjugated antiserum) as described above. When using pre-absorbed sera, confocal imaging settings were identical to those used with the non-absorbed sera.

## Results

**Fig 1:**
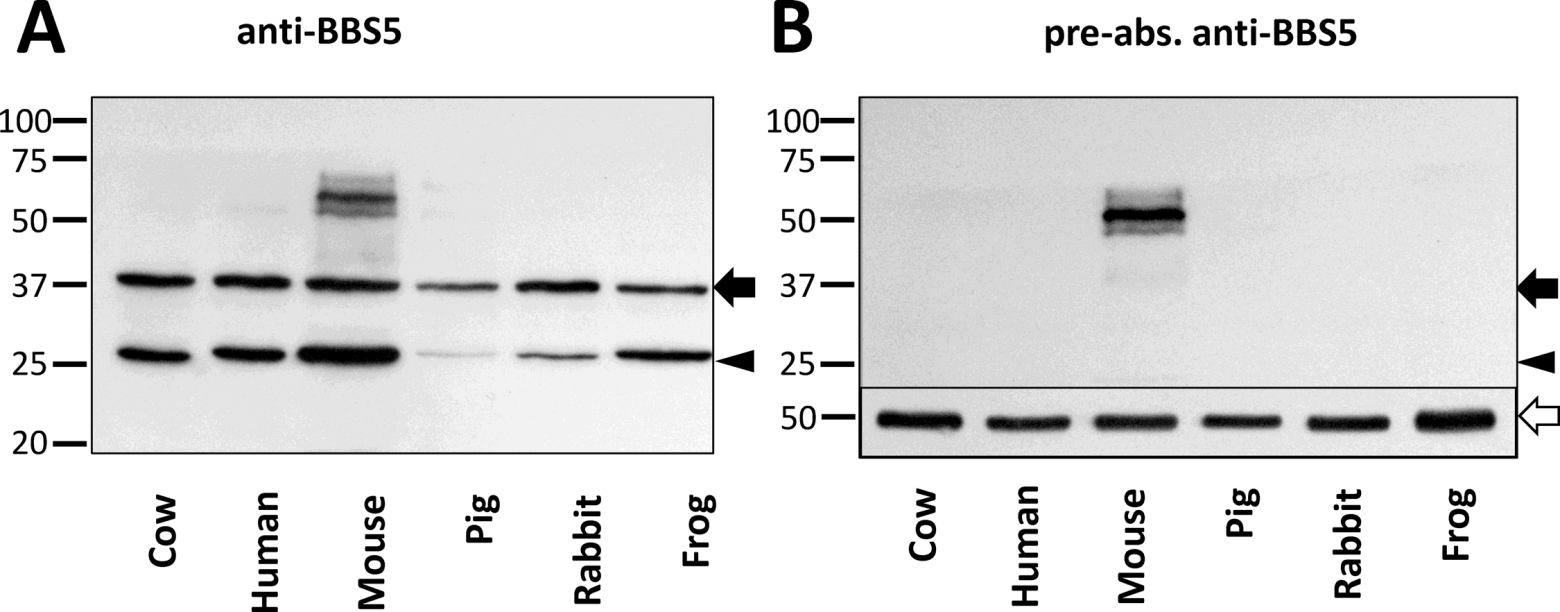
A smaller molecular mass form of BBS5 is detected in retinal extracts from a variety of vertebrates. (A) Immunoblots of extracts of aqueous soluble proteins prepared from whole retinas from the indicated species probed with anti-BBS5 #7–15 monoclonal antibody described in [19]. Arrow indicates the expected molecular size of BBS5; arrowhead indicates a smaller immunoreactive band at 26 kD. (B) A replicate blot was probed with the same antibody that was pre-incubated with heterologously-expressed recombinant murine BBS5. Inset, at the bottom of the blot shows a replicate blot stained for beta-tubulin as a loading control (beta-tubulin indicated with an open arrow).

In the course of our investigations of the role of BBS5 in photoreceptors, we consistently noted on immunoblots that a second, smaller molecular weight band was detected regardless of which anti-BBS5 antibody we used. To investigate this observation more thoroughly, we performed an immunoblot of aqueous-soluble retinal extracts from a variety of species, probing for BBS5 reactivity with an anti-BBS5 monoclonal antibody (Fig 1A). In all six species that we examined, two prominent immunoreactive bands were noted at 38 kD (the expected size for BBS5) and at 26 kD. Reaction with both bands disappeared when the antibody was pre-absorbed with heterologously expressed BBS5 prior to performing the immunoblot (Fig 1B). A higher molecular weight immunoreactive band at ~60 kD present in the mouse sample is likely a non-specific reaction since it is also retained in the blot probed with the pre-absorbed antibody.

**Fig 2:**
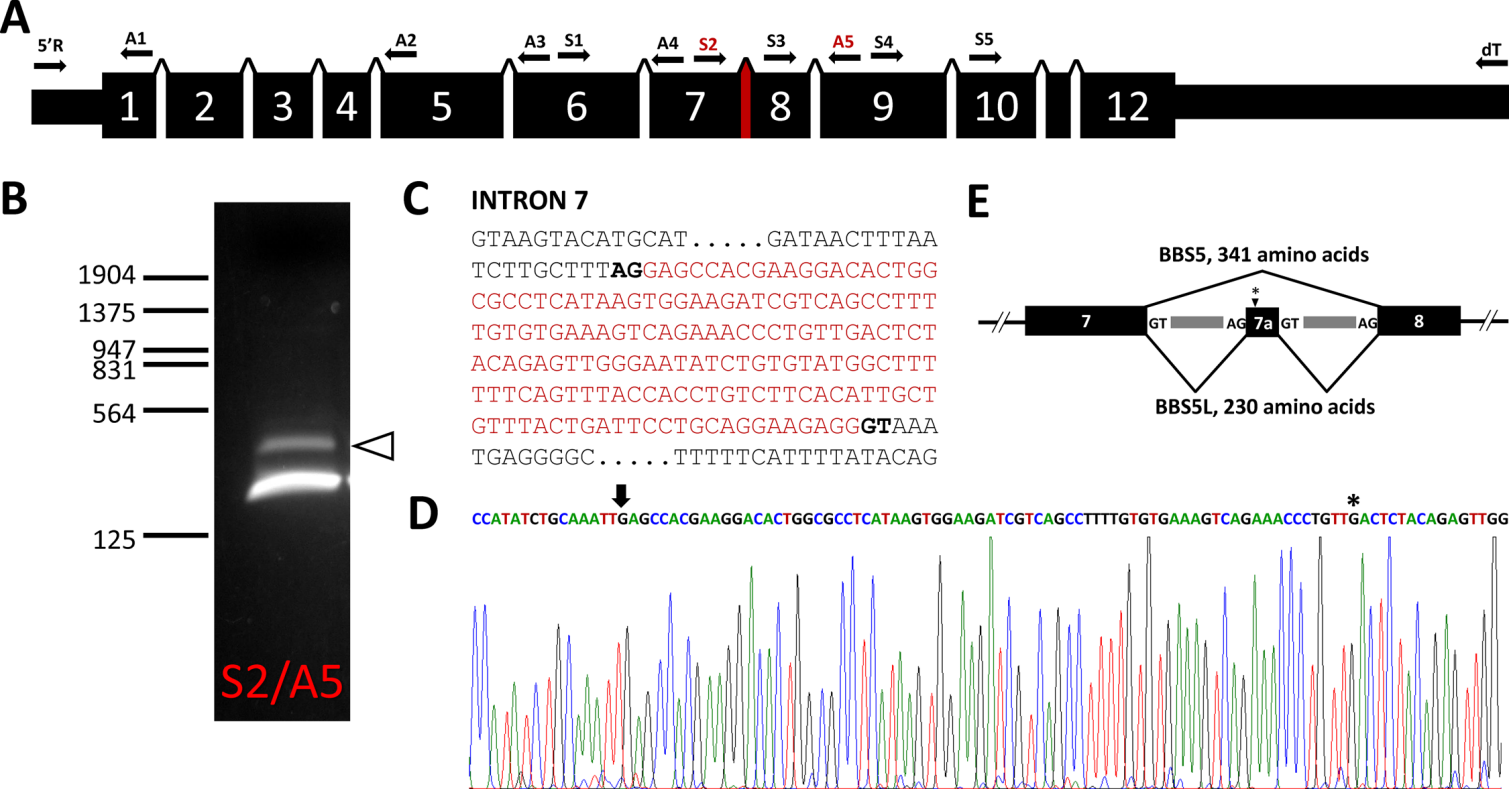
The mouse Bbs5 gene and alternative transcripts. (A) Structure of the murine *Bbs5* gene; locations for sense (S) and anti-sense (A) primers used to test for alternative transcripts are indicated above the gene structure, along with oligo(dT) and 5’RACE primers (5’R). (B) PCR amplification of reverse-transcribed murine retinal poly(A)^+^ RNA with S2 and A5 primers separated by agarose electrophoresis; arrowhead indicates a DNA product larger than the expected 216 bp product. (C) DNA sequence of intron 7 of *Bbs5* with the alternatively spliced region indicated in red (potential splice donor/acceptor sites are bold faced). (D) Sequence chromatogram of the alternative Bbs5 transcript (BBS5L) with the beginning of the cryptic exon 7a (arrow) and stop codon (asterisk) indicated. (E) Gene structure of exons 7, 7a, and 8 showing the two splicing patterns detected for the Bbs5 transcript (the location of the stop codon in Exon 7a is indicated with an asterisk).

This observation suggested that the smaller molecular weight band could potentially be either a variant produced by alternative splicing of the Bbs5 transcript or a proteolysis product of BBS5. To investigate the possibility of alternative splicing, we performed PCR on cDNA prepared from poly(A)^+^ RNA isolated from mouse retina, pairing various sense and anti-sense primers targeted to different exons of the Bbs5 sequence and with oligo(dT) and a 5’ anchor primer generated for 5’ RACE (Table 1; Fig 2A). In nearly every pairing, only the expected Bbs5 was amplified. When we utilized primers S2 against sequence in exon 7 paired with anti-sense primer A5 in exon 9, however, we amplified two bands—the expected 216 bp product and a larger amplification product (Fig 2B). Sequencing of this larger product revealed it to contain a potential cryptic exon located in intron 7 that is flanked by possible splice donor/acceptor sequences (Fig 2C and 2D). Conceptual translation of this product reveals that the exon 7a sequence creates a reading frame shift that terminates after 24 amino acids, offering a potential explanation that this longer transcript, termed Bbs5L, with its shortened C-terminus might be the source of the smaller anti-BBS5 immunoreactive product we detected on blots.

**Fig 3:**
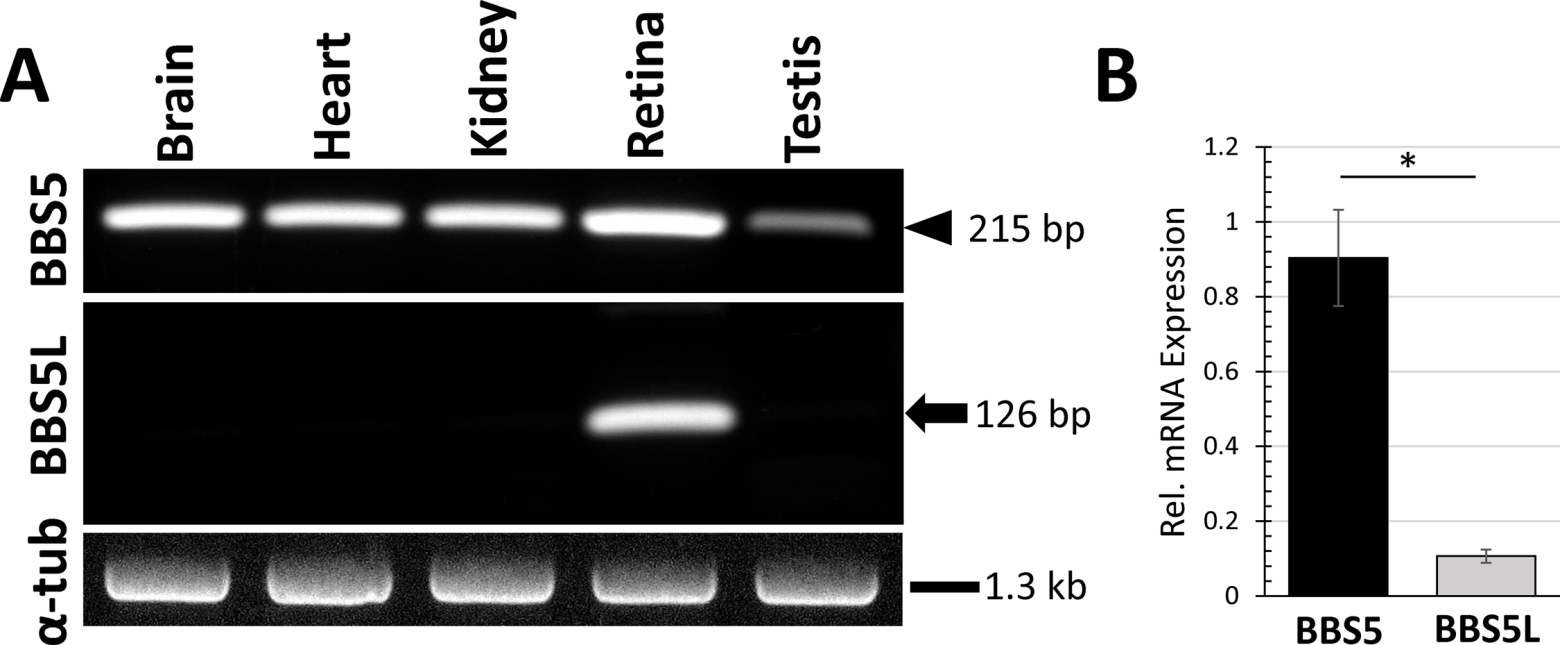
Reverse transcription-PCR amplification of Bbs5 and Bbs5L from various murine tissues. (A) cDNA prepared from poly(A)^+^ RNA isolated from the five indicated tissues was amplified with primers specific for Bbs5 (upper panel; arrowhead indicates expected 215 bp product), Bbs5L (middle panel; arrow indicates expected 126 bp product), or alpha-tubulin for quality control (lower panel). (B) Quantitative RT-PCR was performed for Bbs5 and Bbs5L using cDNA prepared from retinal poly(A)^+^ RNA and quantified relative to levels of β-actin cDNA (n = 3; error bars indicate SEM; ρ<0.05).

To determine the tissue expression of this potential splice variant, poly(A)^+^ RNA was isolated from six different organs from mouse, reverse transcribed with oligo(dT), and then amplified with primers specific for Bbs5 and for the alternative splice variant (Fig 3A). The splice variant of Bbs5 was detected only in RNA isolated from retina, and not from brain, heart, kidney, or testis. Quantitative RT-PCR was performed on retinal poly(A)^+^ RNA, and showed the abundance of the Bbs5L splice variant RNA relative to Bbs5 to be 8.9-fold (+/- 1.1 fold) less than the Bbs5 transcript (Fig 3B).

**Fig 4:**
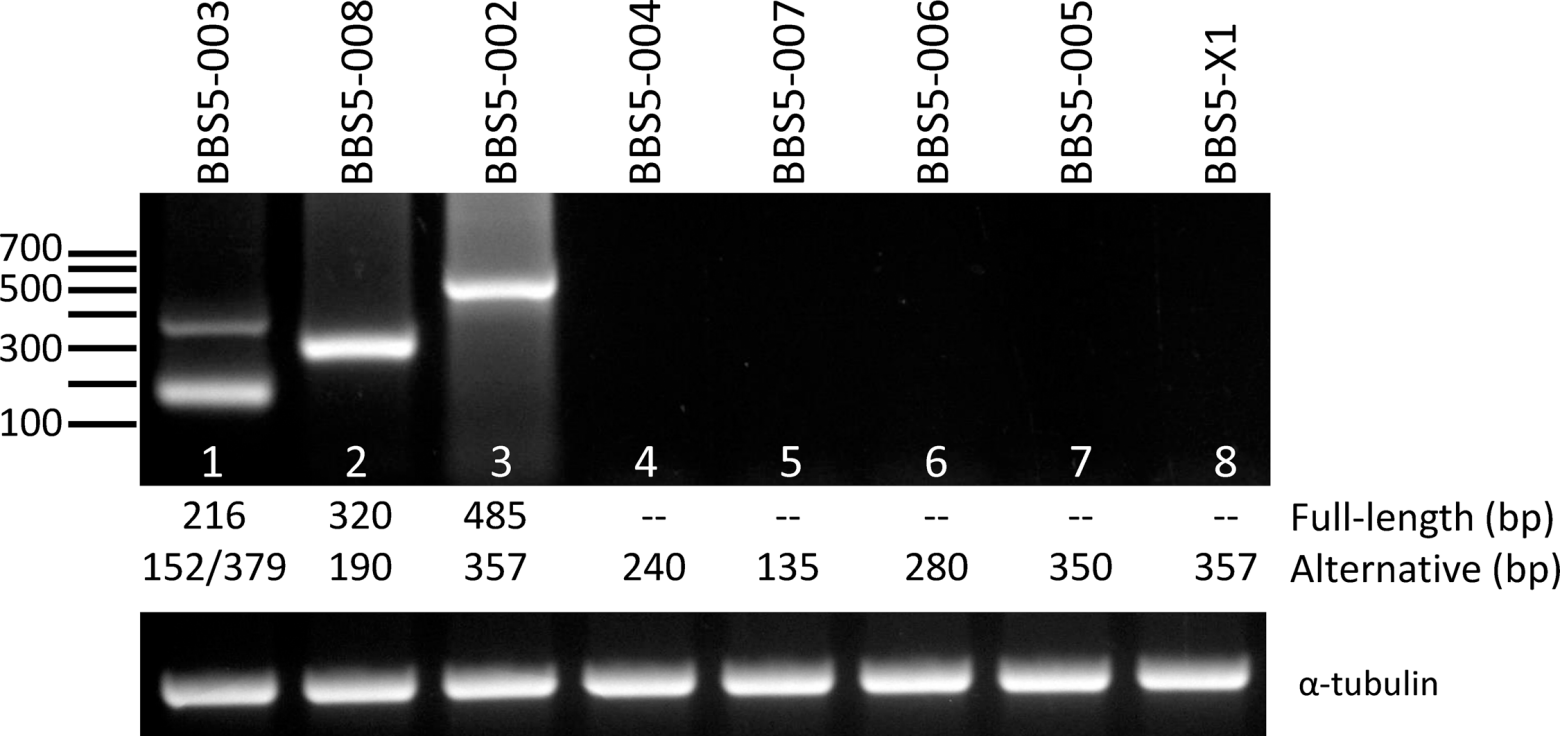
RT-PCR of predicted Bbs5 transcripts from retinal cDNA. The alternative transcripts for mouse Bbs5 predicted in the Ensembl (Bbs5-002 through -008) and MGI (Bbs5-X1) were amplified with primers that would selectively identify the alternative transcript. The predicted sizes (in bp) for each transcript are indicated below each lane for the full-length transcript and the alternative splice variant transcript [two alternative transcripts are possible in lane 1, corresponding to the deletion of Exon 8 (152 bp) or the inclusion of Exon 7A (379 bp)]. Lower panel shows amplification reactions with primers against alpha-tubulin to control for cDNA quality.

Investigation of potential murine Bbs5 transcripts in the Ensembl [25] and MGI [26] databases revealed that this alternative transcript is one of ten potential transcripts predicted to be generated from the murine gene. To determine whether any of these other transcripts might also be expressed in the retina, we designed primers that either flanked a deleted exon or was included in the unique sequence of a retained intron. Table 2 provides a listing of the potential transcripts, including the origin of the alternative splice, along with the primers and predicted product size for the primer pairs. RT-PCR was performed using retinal poly(A)^+^ RNA, and in all cases, the only transcript that was identified was either the full-length Bbs5 transcript or the Bbs5L transcript (lane 1, 379 bp product) (Fig 4). This finding suggests that the Bbs5L transcript is uniquely retained in the retina compared to the other predicted transcripts.

**Fig 5:**
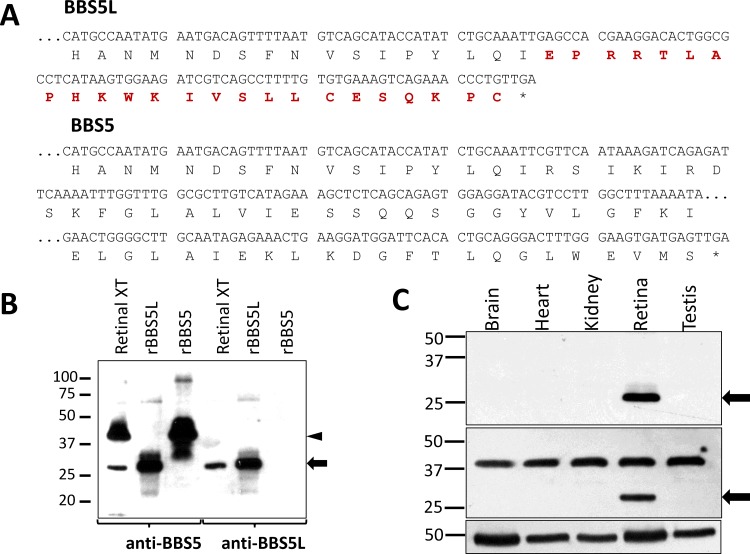
Characterization of a polyclonal antibody specific for BBS5L. (A) Conceptual translation of the alternative Bbs5 transcript (Bbs5L) in comparison with Bbs5 (the unique C-terminus is colored in red). (B) Immunoblots of murine retinal extracts and heterologously expressed BBS5L (rBBS5L) and BBS5 (rBBS5) were probed with either anti-BBS5#7–15 monoclonal antibody (left half) or with anti-BBS5L polyclonal serum (right half); arrowhead indicates BBS5 or rBBS5, arrow indicates BBS5L or rBBS5L. (C) Immunoblot of aqueous-soluble extracts prepared from the indicated mouse tissue probed with anti-BBS5L polyclonal serum identifies anti-BBS5L immunoreactivity only in the retinal extract (upper panel; arrow); an antibody that recognizes both the BBS5 and BBS5L reacts with BBS5 in all extracts, and BBS5L only in retinal extract (middle panel); inset below the blot shows a replicate blot of the tissue extracts probed with anti-beta-tubulin as a loading control.

To further our investigation of the Bbs5L transcript, and since many splicing transcripts are generated from a gene but are not translated, we next examined if the Bbs5L splice variant transcript we identified above is transcribed into a stable protein. To accomplish this goal, we prepared a polyclonal antiserum in rabbit against a peptide synthesized to match the unique 24 amino acid C-terminus of the conceptually translated splice variant (Fig 5A). Validation of the specificity of this antibody was performed by immunoblot, probing blots of heterologously expressed BBS5 and BBS5L (Fig 5B). The anti-BBS5L serum reacted strongly with the recombinant BBS5L protein, but showed no reactivity against the heterologously-expressed BBS5. These findings indicate that the anti-serum is specific for the splice variant form of BBS5. When retinal extract is probed with this same antiserum, a 26 kD band is detected that corresponds to the lower band detected by the pan-BBS5 antibody. This result indicates that the splice variant transcript is translated into a stable protein product in retina. Furthermore, the reaction of this antibody that was raised against the unique C-terminal peptide of BBS5L with the same band identified in Fig 1 offers convincing evidence that the 26 kD band is the product of the alternative transcript. To assess the extent of tissue expression of BBS5L, protein blots of aqueous-soluble extracts from mouse brain, heart, kidney, retina, and testis were probed with the splice variant antiserum (Fig 5C). The BBS5L splice variant protein was only detected in extracts prepared from retina (upper panel), whereas an anti-BBS5 antibody recognizes BBS5 in all tissue extracts, and BBS5L only in retinal extract (middle panel). These immunoblot findings are in agreement with the RT-PCR results.

**Fig 6:**
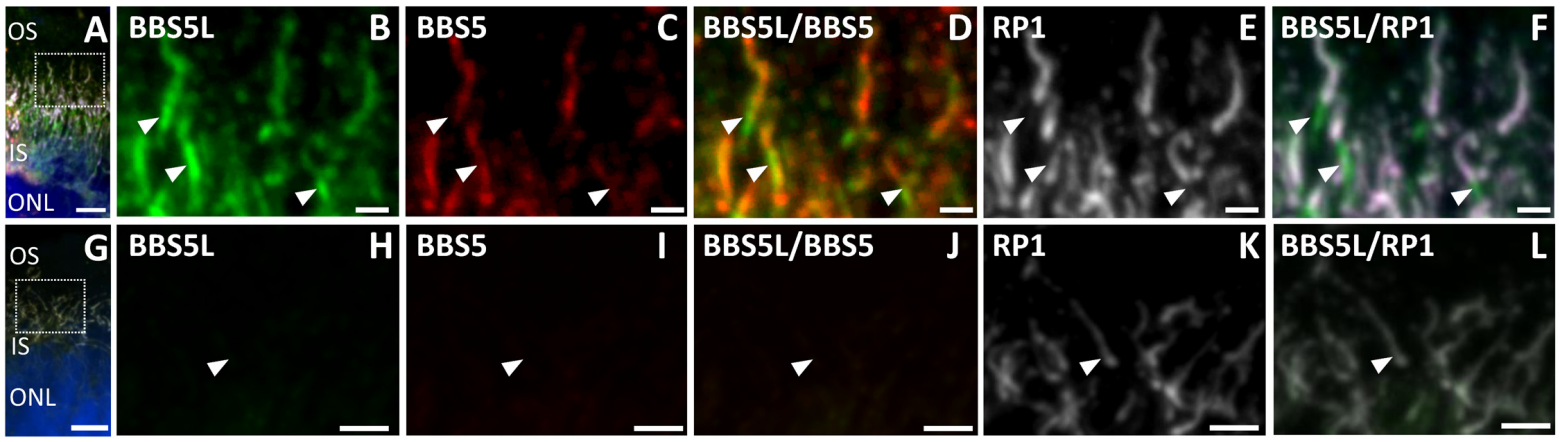
BBS5L localizes to the axonemal structure of the photoreceptors. (A) Lower magnification view of indirect immunofluorescence of antibodies against BBS5L (green), BBS5 (red), RP1 (white), and DAPI (blue) on cryofixed mouse retinal tissue (see methods for specific details on antibodies). (B-E) Enlargement of region indicated in (A) showing localization of BBS5L (B) and BBS5 (C). BBS5L and BBS5 reactivity are highly overlapping as seen in the merge (D). Staining with the axonemal marker RP1 (E) shows that BBS5L co-localizes with RP1 in the distal axoneme (F), but also stains the proximal transition zone of the axoneme where RP1 is absent (white arrowheads). G-L shows parallel staining with pre-absorption controls, using anti-BBS5L antiserum preabsorbed against BBS5L protein (H) or anti-BBS5 antibody preabsorbed against BBS5 protein (I). OS, outer segments, IS, inner segments, ONL, outer nuclear layer; scale bars are 10 μm (A) and 2.5 μm (B-F).

We next examined the expression pattern of the splice variant of BBS5 relative to full-length BBS5 to determine if the BBS5L protein was uniquely distributed in the retina. Sections of cryofixed mouse retina were probed with the anti-BBS5L antiserum and with a polyclonal anti-BBS5 antibody that recognizes both BBS5 and BBS5L (Fig 6). BBS5L immunoreactivity was prominent over the interface region between the outer and inner segments of the photoreceptors (Fig 6A), corresponding to the connecting cilium region. In this staining, there is nearly perfect co-localization of the BBS5L and BBS5 fluorescence (Fig 6D). Since we do not have an antibody that uniquely recognizes BBS5 over BBS5L, however, the only conclusion that we can reach from this co-localization of BBS5 and BBS5L is that BBS5L is not uniquely distributed in any subcellular space that is not occupied by BBS5. The converse cannot be excluded. Co-staining with a axoneme-specific antibody against RP1 [24] shows that the BBS5L staining is confined to the axoneme of the photoreceptors, although the distribution of BBS5L also includes the basal body/transition zone region of the axoneme where RP1 is not localized (Fig 6E & 6F). Pre-absorption of the anti-BBS5L antiserum against purified BBS5L protein and anti-BBS5 antiserum against purified BBS5 protein resulted in the loss of axonemal staining, indicating specificity of the antibodies for the target protein (Fig 6G–6L).

**Fig 7:**
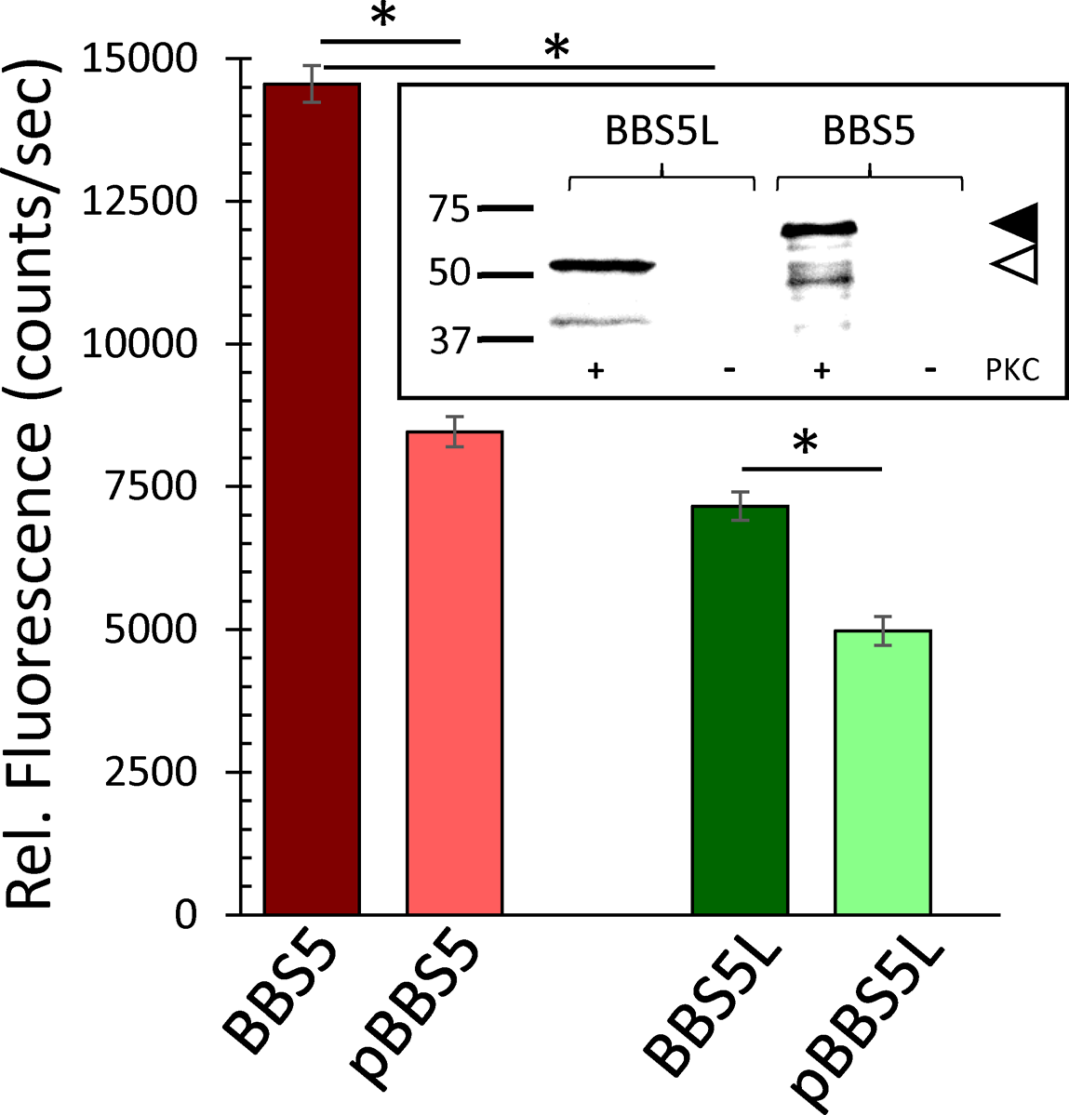
Binding of arrestin1 by BBS5L relative to BBS5, comparing unphosphorylated and phosphorylated conditions. ARR1-Alexa546 was co-immunoprecipitated with BBS5/GST or BBS5L/GST fusion protein with anti-GST antibody and precipitated arrestin1 measured fluorimetrically. ARR1-Alexa546 was pulled down by both BBS5L and BBS5, although the precipitation by BBS5L/GST was significantly less than by BBS5 (n = 6; ρ<0.05). Phosphorylation significantly reduced the pulldown of arrestin1 by both BBS5 and BBS5L (n = 6; ρ<0.05); error bars indicate SEM. Inset shows an immunoblot of the BBS5 and BBS5L proteins probed with anti-phosphoserine antibody after phosphorylation with (+) or without (-) PKC; arrowheads indicate the expected molecular mass for BBS5L-GST (open arrowhead) and BBS5-GST (black arrowhead).

Currently, our knowledge of what BBS5 is doing in photoreceptors is quite limited. Presumably, BBS5 functions as one subunit in the BBSome in what is considered to be the traditional role of the BBSome for controlling the composition of the cilium either through import control [27–30] or export control [13,14] of the proteins entering and exiting the cilium. In terms of potential interacting partners, our recent study of BBS5 indicates an axonemal localization for BBS5 where it binds arrestin1 in a phosphorylation-dependent manner [19]. To determine if the splice variant of BBS5 also binds arrestin1, we heterologously expressed BBS5 and BBS5L in fusion with glutathione-S-transferase, and then immunoprecipitated the BBS5-GST or BBS5L-GST to pull down fluorescently-labeled arrestin1 (Fig 7). Our findings showed that both BBS5 and BBS5L bind arrestin1, but that BBS5L binds only half as much arrestin1 as BBS5 under comparable conditions. Previous studies have shown that the association of arrestin1 with BBS5 is diminished by phosphorylation of BBS5 by protein kinase C (PKC). To test if BBS5L phosphorylation affects binding to arrestin1, we repeated this immunoprecipitation assay using BBS5L that was phosphorylated by PKC. Like BBS5, BBS5L is phosphorylated by PKC (Fig 7, inset), indicating that the altered C-terminus of BBS5L does not affect the PKC phosphorylation sites. In terms of binding to arrestin1, phosphorylation of BBS5L reduces the pull down of arrestin1 by 31%, a smaller decrease than the 42% reduction in arrestin1 pulldown by phosphorylated BBS5 compared to unphosphorylated BBS5. The ability of BBS5L to pulldown arrestin1, and its sensitivity to phosphorylation, indicate that the general binding domain for interaction with arrestin1 is retained in the BBS5L protein. However, the decrease in pull down of arrestin1 suggests that the different C-terminus of BBS5L alters the biophysical properties of the splice variant, likely influencing one or more binding parameters such as affinity, on-rate, or off-rate.

## Discussion

The principal finding from this study is that the *Bbs5* gene is expressed as two stable protein products produced by alternative splicing in a broad range of vertebrates, including mice, cows, humans, rabbits, pigs, and frogs. In mice, the Bbs5 splice variant results from the use of cryptic splice sites in intron 7. This incorporation of exon 7a results in a shift in the open-reading frame and a unique carboxy-terminus. Among the tissues tested that are rich in cilia, the splice variant transcript and protein product were detected only in the retina. Our investigations were unable to discern any unique distribution for BBS5L, compared to BBS5, showing that both proteins are localized to the axonemal structure of photoreceptors. Further, in terms of potential functional interactions, we showed that BBS5L is also capable of binding arrestin1, a previously identified binding partner for BBS5 along the photoreceptor axoneme.

It is curious that the splice variant transcript of Bbs5 identified in this study is the type of transcript which is usually removed by the nonsense-mediated decay (NMD) pathway [31–34]. The inclusion of exon 7a generates two factors that are generally thought to activate NMD—an open-reading frame interruption and a long 3’ UTR [31]. However, the selective expression of the BBS5 splice variant protein in retinal tissue suggests that perhaps the retina offers a unique environment that is permissive for this splice variant. Recently, Nickless et al. suggested that high calcium can inhibit the NMD pathway [35]. Since photoreceptor cells have high calcium levels in the dark (250 nM; [36]) and have intracellular calcium buffer stores to prevent calcium levels from dropping during prolonged light [37], perhaps this offers an explanation as to how Bbs5L is selectively expressed in the retina. However, the fact that other potential transcripts that are predicted from the *Bbs5* gene are not detected in the retina argues that the Bbs5L transcript is somehow unique and perhaps plays a significant functional role in photoreceptors.

Our observation of a retina-specific splice variant for Bbs5 is not unique amongst the Bardet-Biedl syndrome-causing genes. In humans, *BBS8* has an alternative transcript, BBS8L, formed from the incorporation of exon 2a that occurs only in the retina [38] and is nearly exclusively expressed in rod and cone photoreceptors [39]. This particular splice variant was identified because a splice site mutation that eliminates the incorporation of this alternative exon results in retinitis pigmentosa [38]. In considering the known causes of Bardet-Biedl syndrome associated with defects in *BBS5*, most of the disease-causing mutations are associated with the first six exons or introns of *BBS5* [40–42], and these would thus not selectively impact the BBS5L variant. However, there is one Bardet-Biedl syndrome family with a duplication in Exon 12 [43] and one known family with an R207H variant (beginning at Exon 8) that appears to have a modifying effect on the Bardet-Biedl syndrome phenotype [42]. Both these mutations would have no influence on the BBS5L variant and yet still result in Bardet-Biedl syndrome. This observation indicates that a normal BBS5L protein is insufficient to counter the effects of these two *BBS5* mutations. Thus, in contrast to *BBS8*, where there is a clear association of BBS8L with retinitis pigmentosa, there is currently no known pathology that can be ascribed to BBS5L identified in this study.

In addition to splice variants of BBS8, a splice variant of BBS3 has also been identified in humans that is preferentially expressed in retina [44]. Like our splice variant of Bbs5, human BBS3L is formed by the inclusion of a cryptic exon leading to a reading frame shift that generates a unique C-terminus. Selective knockdown of the bbs3L transcript in zebrafish does not have profound effects on the retina, compared to bbs3 knockout, but does lead to disorganization of the inner segment architecture [44].

These three examples of splice variants among the relatively small family of BBS proteins are a reflection of the growing appreciation that the retina is relatively unique in terms of expressing a large number of alternative transcripts. Several studies have shown that compared to other tissues, the retina has an exceptional number of alternative transcripts [45], with nearly a quarter of the retinal genes expressing alternative transcripts [46]. These alternative transcripts are particularly highly expressed during development, especially among retina-enriched genes [47]. These studies, coupled with our identification of an alternative Bbs5 transcript, highlight the importance of future studies targeted at understanding not only the mechanism regulating the expression of the alternative transcripts, but more importantly understanding the functionality of their protein products.
